# Towards a multi-level family resilience promotion framework: a meta-analysis of factors associated with family resilience in families of autistic children

**DOI:** 10.1186/s40359-026-05162-5

**Published:** 2026-07-16

**Authors:** Dinghua Jia, Changwu Zuo, Fengyang Xu, Xiaoyu Zhuang

**Affiliations:** 1 Department of Social Work, Hong Kong Baptist University, Kowloon Tong, Hong Kong; 2 School of Humanities, Jinan University, Zhuhai, Guangdong China; 3 Hong Kong Baptist University Shenzhen Institute for Research and Continuing Education, Shenzhen, Guangdong China

**Keywords:** Autism spectrum disorder, family resilience, risk/protective factors, meta-analysis

## Abstract

**Purpose:**

Families raising autistic children usually experience daily difficulties, such as accommodating their children’s unique communication patterns and behavioral expressions. Despite these potential challenges, families can demonstrate resilience by showing effective family functioning. However, research on the factors that may influence family resilience in this population remains limited. This meta-analysis aims to identify and synthesize the risk and protective factors associated with family resilience among families with autistic children.

**Methods:**

We conducted a systematic review of studies based on six online English databases (PubMed, PsycINFO, Embase, Web of Science, MEDLINE, and Scopus) and two Chinese databases (CNKI and Wanfang). Combined Pearson correlations were calculated by using the random effects model. A moderator analysis was conducted to explore the impact of moderators such as the study region and study quality. The risk of bias and publication bias were also evaluated.

**Results:**

Forty-one studies were examined, where 15 risk and protective factors were identified. Among the 15 factors, 12 are intrapersonal factors (e.g., parental self-efficacy, parental stress, etc.), and 3 are interpersonal factors (social support, family cohesion, and family quality of life).

**Discussion:**

Our findings indicate that the strongest correlations with family resilience were observed among interpersonal factors. In addition, we identified a number of salient (e.g., family cohesion) and contextually unique (e.g., uncertainty about autism) risk and protective factors for family resilience. Building on this, we propose a multi-level family resilience conceptual framework that can inform targeted interventions for families raising autistic children.

**Trial registration:**

This study was registered with PROSPERO (No: CRD42024595923).

**Supplementary Information:**

The online version contains supplementary material available at https://doi.org/10.1186/s40359-026-05162-5.

## Introduction

Autism spectrum disorder (ASD) is a neurodevelopmental condition that begins in early childhood and is characterized by distinct social communication and interaction profiles, as well as restricted, repetitive patterns of behavior and interests [1]. Families raising autistic children frequently face multifaceted life pressures, including financial strain, public stigma, and the demands associated with communication and behavior support needs [2–4]. These challenges can have far-reaching consequences for both individual family members and the family unit, contributing to reduced family functioning, lower family quality of life, and strain on family interactions and relationships [5]. Specifically, behavioral presentations such as aggressive reactions, social withdrawal, and distinct communication patterns can intensify daily caregiving demands and elevate caregiver stress, with downstream effects on family functioning [6]. Comparative evidence also suggests lower overall family quality of life among families of autistic children relative to families of children with attention-deficit/hyperactivity disorder (ADHD) [7].

Despite the difficulties of raising an autistic child, some families show strength and resilience [8]. For example, Bayat found that a number of these families in her study exhibit resilience, characterized by a sense of family connection and closeness, a positive perception of disability, and spiritual and personal growth [9]. This raises an important question: Why do some families successfully navigate risks and remain well-functioning, while others struggle to cope with adversity? The concept of family resilience provides a crucial analytical framework for understanding how families navigate significant challenges and bounce back from stressful experiences [10, 11].

Family resilience is defined as the dynamic process by which a family operates as a functional unit to withstand and recover from significant adversity [10]. In empirical research, family resilience is operationalized through indicators such as family hardiness and family adaptation [12–14]. Crucially, this adaptive process is understood through the interplay of risk and protective factors. According to the ABC-X theory, family resilience emerges from the interaction of four key components: A (the stressor event), B (the family’s crisis-meeting resources), C (the family’s perception and interpretation of the event), and X (the resulting crisis or adaptation outcome) [15, 16]. Based on decades of practical experience, Walsh has also identified several important categories of protective factors. For example, three important categories of factors are belief systems (e.g., positive outlook, making meaning of adversity), organizational patterns (e.g., flexibility, social and community resources), and communication processes (e.g., clarity and problem-solving) [17].

Family resilience plays a critical role in promoting well-being among families raising autistic children. Research has consistently demonstrated that higher family resilience is associated with a range of positive outcomes for caregivers and children. For caregivers, greater family resilience relates to lower anxiety and depression [18], better coping strategies [19], and increased capacity for stress management [20]. For children, higher family resilience (e.g., collaborative problem-solving and emotional connection) is associated with key behaviors of flourishing, such as remaining calm and regulated when facing challenges [21].

### Risk and protective factors for family resilience in general and autistic families

Extant research has identified a wide array of risk and protective factors that influence family resilience within the general population. As suggested by Walsh, these factors operate across multisystemic and multilevel environments, necessitating a holistic approach to understanding family resilience [22]. At the intrapersonal level, factors such as family members’ mental health, shared belief systems, and self-efficacy are more strongly associated with family resilience [23, 24], whereas low socioeconomic status is associated with challenges to family resilience [25]. At the interpersonal level, social support within the family system, collaborative problem-solving, and supportive relationships have consistently been identified as protective factors for family resilience [25].

In families of autistic children, several important risk and protective factors for family resilience have been identified. On the intrapersonal front, adaptive coping strategies and improved parental mental health serve as core protective factors for family resilience, while parental stress—often compounded by the need to accommodate autistic children’s repetitive behavioral traits and unique social communication patterns—is negatively correlated with family resilience [26, 27]. At the interpersonal level, accumulating evidence suggests that robust social support networks, particularly from extended family and community resources, are a key protective factor for family resilience among this group of families [28].

While families raising autistic children share certain protective factors with the general population, meaningful differences in their resilience profiles remain. These differences stem from the unique contextual constraints and structural stressors inherent to caring for autistic children, including the sustained demand for specialized interventions, challenges related to the child’s distinct social communication and interaction patterns, and the significant emotional and financial impact experienced by caregivers [9]. Such distinct stressors suggest that specialized formal and informal support—ranging from professional guidance at autism-specific rehabilitation centers to emotional assistance from extended family—may exert a far more significant and tailored impact on family resilience than generalized social support, which may not address the unique needs of this group [29]. Consequently, conclusions regarding protective factors derived from general population research cannot be readily generalized to this specific group [30]. Accordingly, a systematic understanding of the risk and protective factors for family resilience among families of autistic children, along with identifying the factors most strongly correlated with family resilience, is crucial for guiding the design and implementation of targeted interventions.

### Research gaps

Although research has identified certain risk and protective factors for family resilience, several key limitations remain. First, the context-specific nature of family resilience is insufficiently addressed. Existing literature demonstrates that the risk and protective factors for family resilience may vary significantly across different populations and circumstances [31]. Factors identified in the general population may not confer comparable benefits for families raising autistic children. These families often confront distinctive and enduring challenges—including social stigma, fragmented and complex service systems, and ongoing demands associated with core autism characteristics [32]. Despite these challenges, few studies have systematically examined which risk and protective factors are most salient for autistic families or how interventions can be tailored to their needs.

Second, to date, no meta-analysis has been conducted on family resilience among families of autistic children. To our knowledge, only one systematic review has examined this literature [33]. While this review identified relevant stressors (e.g., sleep disturbances, uncontrollable behaviors), protective resources (e.g., formal support, strong family cohesion), and meaning‑making processes (e.g., positive reframing) linked to resilience, it did not include a quantitative meta‑analysis. As a result, the relative magnitude and effect sizes of these diverse risk and protective factors remain unestablished. This gap hinders the identification of priority targets for intervention and policy development.

Third, current research has not directly compared the relative contributions of interpersonal versus intrapersonal factors to family resilience in families raising autistic children. Most studies examine these domains in isolation or focus predominantly on one, leaving a critical gap in understanding their comparative impact [34]. Without clear evidence about which domain exerts greater influence, it is difficult to design targeted, evidence-based interventions that allocate resources efficiently and maximize support for these families. Clarifying the relative importance is therefore essential to optimize intervention strategies and inform the design of support programs that address this population’s specific challenges. Such evidence would also support domain-specific prioritization, enabling tailored supports for distinct family profiles and guiding policy decisions on allocating scarce training and service resources.

### The present study

To address these gaps, the present study is, to our knowledge, the first meta-analysis to systematically explore risk and protective factors associated with family resilience among families with autistic children. Our primary objective is to identify the risk and protective factors for family resilience by synthesizing the effect sizes of different factors. Additionally, we also explore potential moderators that may influence the impact of each risk and protective factor, including study characteristics (such as study region and publication year) and sample characteristics (such as age and sample size). By doing so, this review will provide a quantitative evidence base to guide future research and support efforts aimed at enhancing family resilience.

## Methods

This systematic review was registered with PROSPERO under registration number CRD42024595923 and follows the Preferred Reporting Items for Systematic reviews and Meta-Analyses (PRISMA) framework [35], thus adhering to established methodological reporting standards.

### Search strategy

Six online English databases (PubMed, PsycINFO, Embase, Web of Science, MEDLINE, and Scopus), and two Chinese databases (CNKI and Wanfang) were used to conduct the systematic review. ProQuest was used to search for dissertations and theses. Studies that were published before November 2024 were considered for inclusion. To incorporate the most comprehensive collection of relevant literature, broad search terms were used, such as “child*”, “autis* OR Asperger* OR autistic disorder OR pervasive developmental disorder” and “family resilienc* OR family hardiness OR family adaptation OR family adjustment”. The search terms and their variations can be seen in the Supplementary Table 2.

### Inclusion/exclusion criteria

We included studies that satisfy these criteria: (1) the participants are the caregivers (over 18 years old) of autistic children; (2) original research work; (3) cross-sectional or longitudinal studies; and (4) the study reported correlations with family resilience for at least one factor.

Studies that have the following criteria were excluded: (1) evaluations of programs that enhance family resilience; (2) non-original research (such as literature reviews); and (3) full-text or final results are not available (such as protocols).

### Risk of bias

Risk of bias assessment evaluates flaws in individual study’s design, conduct, and reporting that may introduce systematic error, in order to judge the credibility of its findings. The Newcastle-Ottawa Scale (NOS), specifically adapted for cross-sectional studies, was employed to evaluate the quality of the included studies [36]. This scale comprises 7 items that evaluate 3 key domains: selection, comparability, and outcomes. The selection domain includes 4 items that evaluate the sample size, sample representativeness, the handling of non-respondents, and ascertainment of risk or protective factors. The comparability domain consists of a single item that examines whether the subjects are comparable across different outcome groups. Finally, the outcomes domain includes 2 items that evaluate the outcome and statistical tests employed. The maximum total score for the NOS is 10, with 5 points allocated for selection, 2 for comparability, and 3 for outcomes. Each study was classified into one of three quality categories: high quality (≥ 8), moderate quality (6–7), or low quality (≤ 5) (see Supplementary Table 3). Two reviewers (DJ and FY) conducted the evaluation independently, and their results showed substantial agreement (Cohen’ *κ* = 0.75). Any discrepancies were addressed through discussion.

### Data extraction and coding

After the search, a full-text review was conducted to find the relevant publications, and the two reviewers independently evaluated the titles and abstracts. Data were extracted on 23 January 2025. Two reviewers independently collected the following information: author(s), publication year, country of the study, age, female ratio, autism status, sample size, scales for family resilience, risk/ protective factors, category, and quality score. The effect size was extracted based on these criteria: (1) zero-order correlation coefficients were reported without any adjustment for covariates, and (2) odds ratios and *β* values were converted to *r*.

Risk and protective factors were identified in the data extraction stage. Notably, a substantial body of prior resilience research has adopted the social‑ecological framework to examine risk and protective factors, and categorizing factors via this multi‑level lens can improve theoretical organization and cross‑study comparability [31, 37]. Therefore, guided by the Social Ecological Model (SEM) [38], factors were classified into intrapersonal and interpersonal levels. Intrapersonal factors refer to individual-level characteristics (e.g., demographic factors, biological factors, psychosocial constructs such as self-efficacy, knowledge, attitudes, beliefs, skills, and capabilities), while interpersonal factors involve relationships and interactions with family, peers, partners, and social networks. Two independent coders completed a two-hour training on these definitions and categorization criteria. They then piloted the coding on five pre-screened studies to align interpretations and refine category boundaries, using illustrative variables: parental stress and parental self-efficacy for the intrapersonal category, and family cohesion and social support for the interpersonal category. A unified coding template was applied to all studies, recording study ID, extracted variables, the intrapersonal/interpersonal classification, and the rationale for each coding decision. Discrepancies in data extraction and coding were first addressed through consensus discussions, with each coder articulating their rationale; unresolved cases were adjudicated by an independent third expert, ensuring methodological rigor and transparency throughout the review. We contacted three of the authors for further information, but none of them responded to the request for the data.

### Meta-analytic procedures

In this study, random effects models were employed to combine the effect size, given the considerable heterogeneity between studies for most of the factors. The effect size was measured by using Pearson’s correlation coefficient (*r*). For the studies that provided standardized *β* coefficients, we transformed the *β* to *r* based on the equation in Peterson and Brown: *r* = .98 *β* + 0.05 *λ*, where *λ* = 0 when *β* < 0; *λ* = 1 when *β* ≥ 0 [39]. For the studies that provided odds ratios (OR), ORs were transformed into *r* via the Rosenthal formula: *r* = $\:\frac{\sqrt{OR}-1}{\sqrt{OR}+1\:}$ [40]. We adopted these widely acknowledged conversion procedures to ensure comparability of effect sizes in the present meta-analysis. All of the correlation coefficients were transformed by using Fisher’s *r*-to-*z* method to achieve normally distributed *z* values, which allows for the calculation of 95% confidence intervals [41]. According to Cohen’s criteria, effect sizes of 0.1, 0.3, and 0.5 are classified as small, medium, and large, respectively [42]. The meta-analysis pooled risk or protective factors for family resilience from at least two or more studies.

We also tested for and defined heterogeneity levels across studies with *I*^*2*^ values of 25%, 50%, and 75%, which are low, moderate, and high degrees of heterogeneity, respectively [43]. Publication bias assessment determines whether selective publication or reporting across studies systematically skews the evidence, causing pooled effect estimates to misrepresent the totality of conducted research. To assess the publication bias, we conducted Egger’s tests [44]. Moreover, a range of moderator analyses were performed to account for the variation in effect sizes by using mixed effect models. For continuous moderators, such as mean age, publication year, and sample size, we conducted meta-regressions. Comprehensive Meta-Analysis software (Version 3.7) was used to analyze the data.

## Results

### Characteristics of the studies

**Fig. 1:**
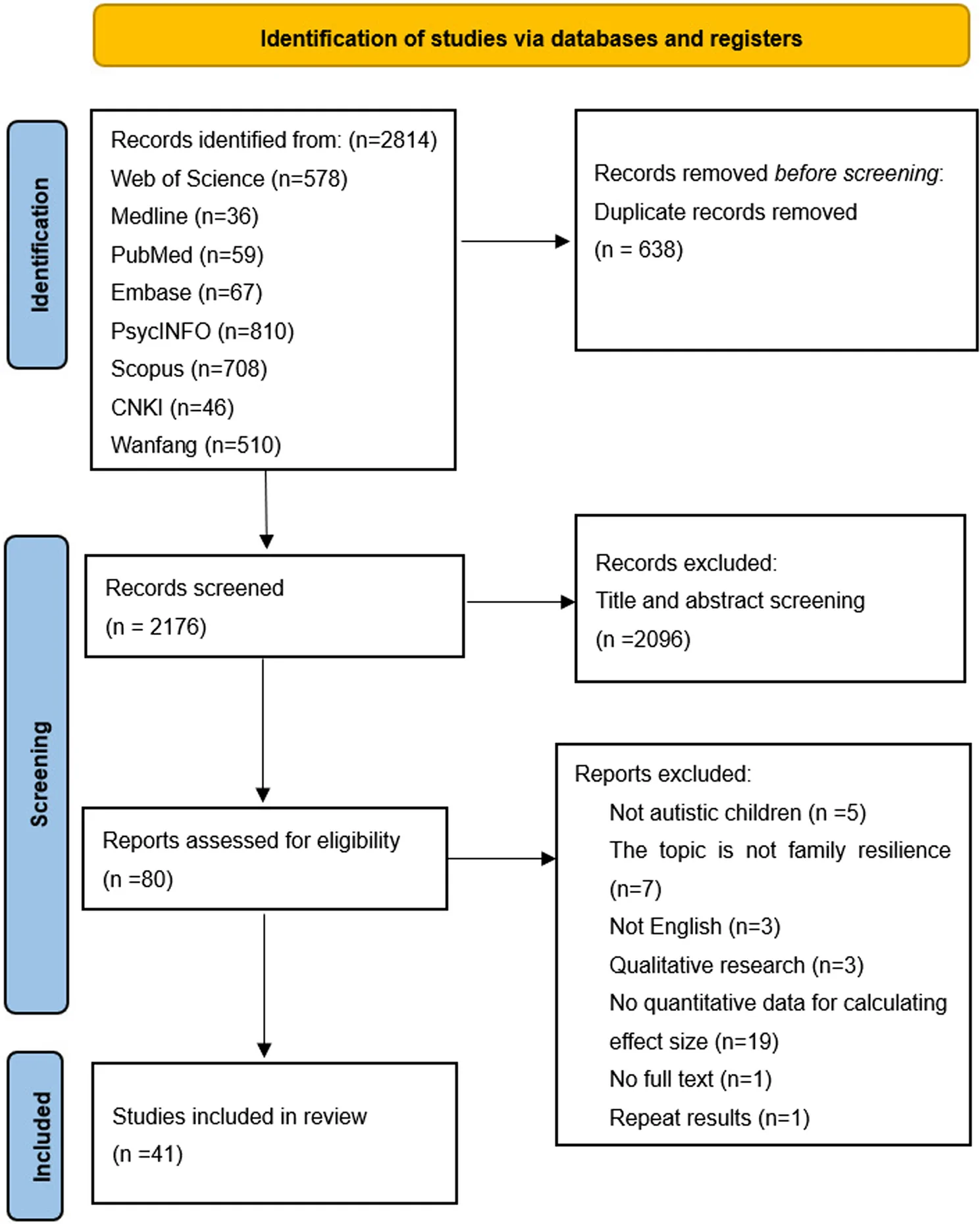
RISMA flowchart of study selection

Figure 1 presents the screening flowchart based on the PRISMA guidelines. In total, 2814 references were found, which include both English and Chinese articles. After removing duplicate records, 2176 entries remained for further screening. After an initial screening of the titles and abstracts, 80 full-text articles were retrieved and evaluated for eligibility. Among them, five did not focus on autistic children, seven did not pertain to family resilience, three were not in English, three lacked the quantitative data necessary for calculating effect sizes, one was not a full-text article, and one contained duplicate results. Ultimately, 41 articles met the inclusion criteria.

The characteristics of the studies are detailed in Supplementary Table 3. The total sample size across all of the studies is 15,817 participants, with individual sample sizes that range from 34 to 3247 (mean sample size = 386). The age of the participants varies between 19 and 73 years old. Most studies were conducted in the USA (*k* = 19) and China (*k* = 11); others were conducted in Canada (*k* = 4), Ireland (*k* = 2), South Africa (*k* = 2), Australia (*k* = 1), Singapore (*k* = 1), Romania (*k* = 1), and Greece (*k* = 1). The proportion of female participants varied between 50% and 100%. Among the participants’ children, girls accounted for 7.7% to 35%. In terms of study quality, most were rated as having a moderate (*n* = 23; 54.8%) quality, while 12 of the studies (28.6%) were classified as having high quality, and only seven of the studies (16.7%) were deemed low in quality.

### Effect size estimates

**Fig. 2:**
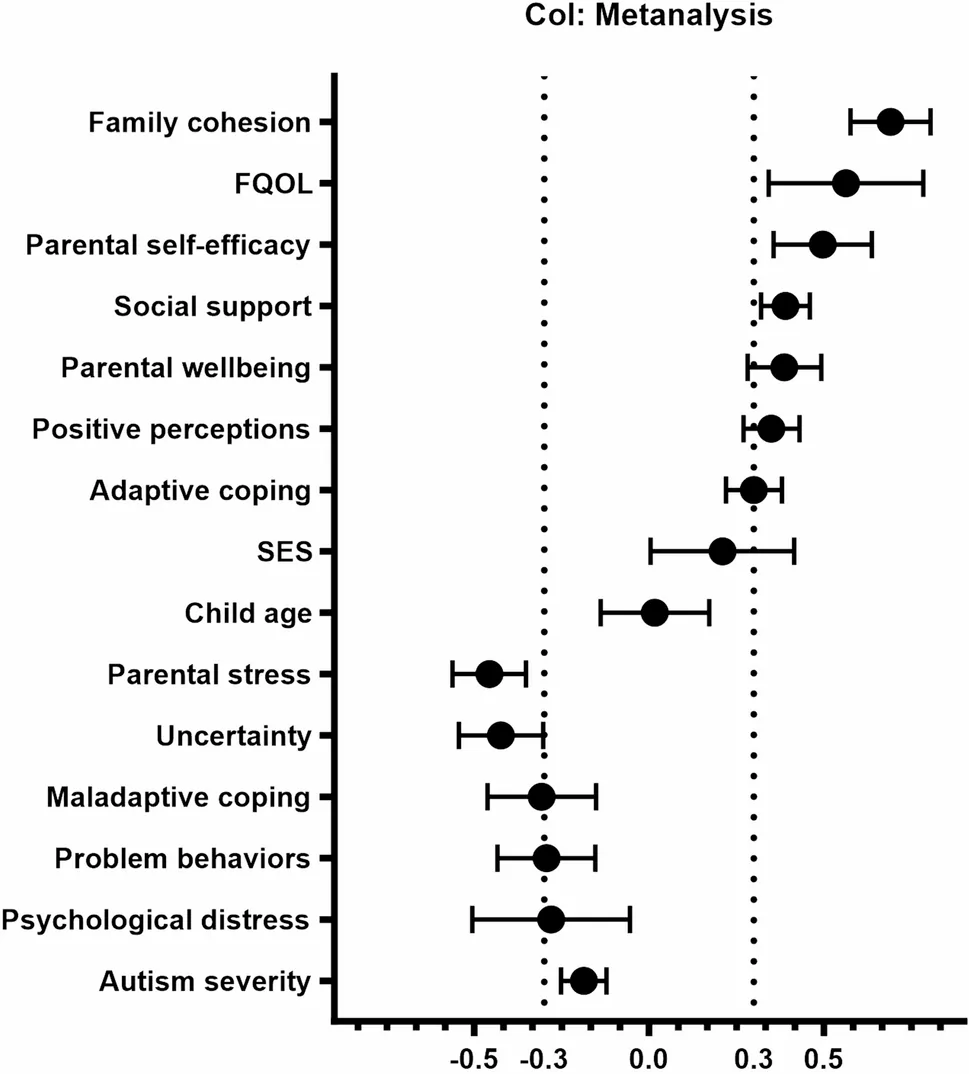
Pooled effect size estimates

The meta-analysis included fifteen risk and protective factors. The primary findings of the meta-analysis are presented in Fig. 2. Effect sizes are shown in descending order, protective factors are listed above, and risk factors are listed below. The factors are grouped into two categories: intrapersonal and interpersonal factors. The key findings for each risk/protective factor related to family resilience are presented in Table 1.

**Table 1:** Pooled effect size estimates, heterogeneity, and publication bias

Risk/ Protective factors	Category	Factors	*N* of studies	Sample size	Effect size *r* [95% CI]	I^2^ (%)	Egger’s test, *p*
Protective factors	Interpersonal factors	Family cohesion	4	609	0.70 [0.57, 0.80]	82.45	0.84
		FQOL	4	526	0.59 [0.33, 0.77]	93.09	0.48
		Social support	20	8907	0.39 [0.32, 0.46]	91.51	0.07
	Intrapersonal factors	Parental self-efficacy	2	274	0.51 [0.35, 0.63]	59.54	NA
		Parental well-being	7	5534	0.39 [0.28, 0.49]	94.42	0.05
		Positive perceptions	4	513	0.35 [0.27, 0.43]	0.00	0.75
		Adaptive coping	13	1866	0.30 [0.22, 0.38]	67.33	0.15
		SES	4	415	0.22 [0.00, 0.41]	75.62	0.82
		Child age	5	424	0.02 [-0.14, 0.17]	55.58	0.36
Risk factors	Intrapersonal factors	Parental stress	11	6698	− 0.46 [-0.56, -0.35]	96.21	0.10
		Uncertainty	2	310	− 0.43 [-0.54, -0.30]	37.00	NA
		Maladaptive coping	6	711	− 0.31 [-0.46, -0.15]	76.00	0.21
		Problem behaviors	7	2825	− 0.30 [-0.43, -0.15]	86.79	0.001
		Psychological distress	6	1159	− 0.29 [-0.50, -0.05]	94.24	0.98
		Autism severity	8	1870	− 0.19 [-0.25, -0.12]	22.17	0.46

#### Intrapersonal factors

Twelve factors were identified in the intrapersonal category, such as parental well-being and parental stress. Among risk factors for family resilience, parental stress showed the strongest negative association (*r* = − .46, 95% CI [-0.56, -0.35]), closely followed by uncertainty (*r* = − .43, 95% CI [-0.54, -0.30]). Maladaptive coping (*r* = − .31, 95% CI [-0.46, -0.15]) and problem behaviors (*r* = − .30, 95% CI [-0.43, -0.15]) also had moderate negative correlation with family resilience. Additionally, psychological distress (*r* = − .29, 95% CI [-0.50, -0.05]) and the autism severity (*r* = − .19, 95% CI [-0.25, -0.12]) were observed to have a small to medium negative association with family resilience.

As for the protective factors, parental self-efficacy (*r* = .51, 95% CI [0.35, 0.63]) was the most significant, followed by parental well-being (*r* = .39, 95% CI [0.28, 0.49]). Positive perceptions (*r* = .35, 95% CI [0.27, 0.43]), adaptive coping (*r* = .30, 95% CI [0.22, 0.38]), and socioeconomic status (SES) (*r* = .22, 95% CI [0.00, 0.41]) also positively correlated with family resilience. However, the effect size for child age (*r* = .02, 95% CI [-0.14, 0.17]) was not statistically significant. The majority of the effects were medium in magnitude.

#### Interpersonal factors

Three factors were classified as interpersonal factors: family cohesion, family quality of life, and social support. The most significant interpersonal factor was family cohesion (*r* = .70, 95% CI [0.57, 0.80]). Following this, family quality of life (*r* = .59, 95% CI [0.33, 0.77]) and social support (*r* = .39, 95% CI [0.32, 0.46]) were found to have large and medium correlations with family resilience, respectively. The meta-analysis indicated that interpersonal factors were of medium to large magnitude.

Among all interpersonal and intrapersonal factors, the heterogeneity analysis indicated that six factors showed low-to-moderate heterogeneity (*I*^*2*^ = 0%–67.33%), whereas nine factors exhibited high heterogeneity (*I*^*2*^= 75.62%–96.21%). The high *I*^*2*^ values reflect considerable between-study inconsistency, indicating the pooled effect sizes may not represent a uniform underlying effect and could be partially influenced by unmeasured study characteristics.

### Moderator analysis

Given the substantial heterogeneity, we carried out moderator analyses for risk and protective factors that were reported in at least 10 studies. Three factors that met the criteria for the analysis, including “social support”, “adaptive coping”, and “parental stress”. The moderators included categorical moderators (such as study region and study quality) and continuous moderators (such as child mean age, publication year, and sample size). The findings from the categorical and continuous moderator analysis are detailed in Supplementary Tables 4 and 5.

Among all associations tested, only one continuous moderator—sample size—significantly moderated the association between parental stress and family resilience (*β* = 0.0002, *p* < .05). Although statistically significant, the regression coefficient for sample size was negligible, indicating that it explained only a minimal fraction of the heterogeneity and held limited practical significance. Furthermore, other potential moderators—including publication year, study region, study quality, and age—did not significantly moderate any of the examined risk- or protective-factor associations, meaning they failed to explain the between-study heterogeneity. This persistent heterogeneity suggests that unmeasured study and sample characteristics, such as variations in measurement instruments or cultural contexts, likely account for the remaining between-study differences.

### Publication bias

Egger’s *p* was used to evaluate publication bias, revealing that problem behaviors exhibited significant publication bias (Egger’s *p* < .05). Since parental self-efficacy and uncertainty were examined in only two of the included articles, it was not possible to calculate the publication bias by using Egger’s *p*. Other risk and protective factors like parental stress, parental well-being, social support, and family cohesion did not show publication bias.

## Discussion

This meta-analysis explored the risk and protective factors associated with family resilience among families with autistic children. To the best of our knowledge, this is the first meta-analysis to address this topic. Across the 41 studies in this paper, we identified 15 risk and protective factors, with effect sizes that vary between − 0.46 and 0.70. Among the interpersonal factors, we observed the largest effect size for family cohesion, followed by family quality of life and social support. Among the intrapersonal factors, parental self-efficacy showed the largest effect size, followed by parental stress, uncertainty, parental well-being, positive perceptions, maladaptive and adaptive coping, problem behaviors, psychological distress, SES, autism severity, and child age. The most interesting finding was the identification of some contextually unique risk factors for family resilience. Among these, uncertainty regarding autism and children’s problem behaviors showed moderate correlational strengths. Another intriguing finding was that the strongest individual associations with family resilience emerged within interpersonal factors (e.g., family cohesion, family quality of life), rather than intrapersonal factors (e.g., parental well-being and coping).

The finding that interpersonal factors (i.e., family cohesion and quality of life) exhibited the strongest correlations with family resilience underscores the value of a systemic perspective. According to Bowen and Minuchin, the family operates as an interconnected system where members mutually influence each other, and through their interactions, have profound and lasting impacts [45, 46]. Henry further posited that the family is a dynamic system that is intricately connected to its environment, which shows the interaction between internal family dynamics and external factors in shaping family resilience [47]. When facing challenges, the collective problem-solving capacity of the family, which effectively integrates the wisdom and resources of the different members, often surpasses the independent ability of an individual to address difficulties. These findings not only enhance the current understanding of the mechanisms that underlie family resilience but also provide the crucial theoretical foundations for family intervention and support strategies.

### Salient interpersonal protective factors associated with family resilience in families of autistic children

Among the identified factors, family cohesion—a key interpersonal factor—emerged as the strongest protective factor for family resilience (*r* = .70). This aligns with Black and Lobo (2008), whose review also identified cohesion as a prominent protective and restorative characteristic of resilient families. Family cohesion refers to the emotional bonds among family members and the balance between closeness and individual autonomy within the family system [48]. Consistent with Walsh’s family resilience model, connectedness and cohesion within the family organization are critical processes for fostering family resilience [10]. Cohesion enhances family resilience by building trust and shared commitment, facilitating open communication and collaborative problem-solving, and enabling flexible role negotiation and mutual support [10]. These processes cultivate collective efficacy, buffer stress, reduce conflict, and help families maintain routines and a stable emotional climate during adversity. In families raising autistic children, higher cohesion can unify caregiving efforts, distribute responsibilities more equitably, sustain positive communication, and support coordinated intervention and advocacy. Together, these mechanisms strengthen the family’s capacity to adapt, cope, and recover in the face of challenges.

Similarly, family quality of life—a construct closely related to cohesion—showed a strong positive association with family resilience (*r* = .59) in our sample. Family quality of life reflects overall family well-being, including the fulfillment of family needs, enjoyment of time together, and the capacity to pursue and achieve goals central to happiness and satisfaction [49]. In the context of autism, higher family quality of life is closely linked to greater resilience, as it not only buffers parental stress through adequate resources and met needs but also strengthens family bonds through positive shared experiences. Furthermore, progress toward valued goals fosters collective efficacy and hope, cultivating a shared sense of meaning. These conditions support open communication, coordinated problem-solving, and stable yet adaptable routines, all of which help families absorb strain, adapt, and recover during adversity. Together, the above two family-level findings suggest that close, flexible relationships, combined with strong and functional organizational structures, are hallmarks of both high family quality of life and family cohesion, and they equip families to adapt and function effectively in the face of challenges [10, 50].

Our study also underscored the crucial role of social support—from communities, neighbors, friends, and peers—as a key correlate of family resilience in families with autistic children (*r* = .39). This outcome aligns with the Resiliency Model of Family Stress, Adjustment, and Adaptation and the family resilience framework developed by McCubbin and Walsh, both of which posit that social support is an essential resource for families navigating crises, such as raising autistic children [10, 51]. Such support not only assists families in coping with immediate challenges but also facilitates the restoration of the pre-crisis equilibrium [52]. A substantial body of evidence has consistently shown that social support is one of the protective factors for family resilience among caregivers of children with developmental disabilities [53] and epilepsy [54]. These findings highlight the importance of promoting strong social support networks aimed at enhancing resilience in families caring for autistic children and other developmental disorders.

### Salient intrapersonal protective and risk factors associated with family resilience in families of autistic children

Moreover, our study identified parental self-efficacy as a strong correlate of family resilience (*r* = .51), a factor not highlighted in prior systematic reviews of family resilience in either general or vulnerable populations. Self-efficacy is the belief in one’s capacity to achieve goals, complete tasks, and overcome challenges [55]. As Bandura noted, individuals with high self-efficacy are more inclined to undertake challenging tasks, persist in the face of adversity, and demonstrate enhanced psychological resilience [55]. In the context of families with autistic children, it is plausible that higher parental self-efficacy co-occurs with greater sustained effort, proactive problem-solving, and effective crisis navigation, all of which are, in turn, associated with stronger family resilience. However, empirical research that directly examines this relationship in the context of autism remains limited. We therefore recommend that future studies use longitudinal or experimental designs to clarify the directionality and potential mechanisms linking parental self-efficacy to family resilience in this population.

Last but not least, parental stress was identified as the largest risk factor in relation to family resilience (*r* = − .46). Research suggests that parents raising autistic children often face immense pressure, including higher financial impact and greater social stigma, and need to deal with the meltdown, stimming, and aggressive behavior of their autistic children, which may impact their emotional and psychological well-being [56]. This stress often makes it harder for parents to recognize their child’s strengths and leaves them with less energy for collaborative problem‑solving, both linked to lower family resilience [57]. These findings underscore the critical need for addressing psychological and parenting stress and improving the well-being of parents. Some clinical trials have shown that mindfulness-based interventions can significantly reduce parental stress and enhance family resilience [58], although causal conclusions remain to be validated by future longitudinal or experimental research.

### Contextually unique findings of risk factors for family resilience among families of autistic children

We identified some unique risk factors that are particularly relevant to families with autistic children. First, the most unique finding in our meta-analysis lay in the identification of uncertainty as a substantial risk factor for family resilience (*r* = − .43). This finding aligns with both Walsh’s theoretical perspectives and existing empirical evidence, highlighting the salience of uncertainty in family resilience [10, 59]. Uncertainty refers to the inability to predict outcomes related to disease events or diagnoses, as well as the implications of these events [60]. For instance, family members may lack sufficient information about autism and have different views on the cause and treatment [61]. In addition, uncertainty may also affect coping strategies. When family members feel uncertain about future health or treatment outcomes, they may adopt avoidance or passive coping styles instead of actively seeking solutions. Passivity not only erodes family resilience but also leads to estrangement and disharmony between family members, which ultimately results in less family resilience [10]. All in all, promoting family resilience among families with autistic children can be effectively done through open communication, addressing uncertainties, and providing clear information.

Second, our results also indicated that problem behaviors and autism severity were correlated with family resilience at low to moderate levels. Sikora found that behaviors such as meltdowns and stimming in autistic children, together with the autism severity, significantly predict lower parental resilience and impact family well-being and functioning [6]. Caring for autistic children can present ongoing demands, and the autism severity, together with behaviors such as aggression or emotional distress, may have a profound influence on the overall well-being of the family unit. For instance, Hodgetts emphasized that some autistic children may engage in aggressive behaviors in certain contexts, which can affect their quality of life and place additional demands on family support systems and resources [62]. These circumstances may contribute to increased parental stress and a reduced sense of competence, thereby compromising family resilience and overall well‑being. This finding highlights the critical significance of early interventions as well as behavioral therapies that aim to provide appropriate support and address these situations.

### Towards a multi-level family resilience conceptual framework

**Fig. 3:**
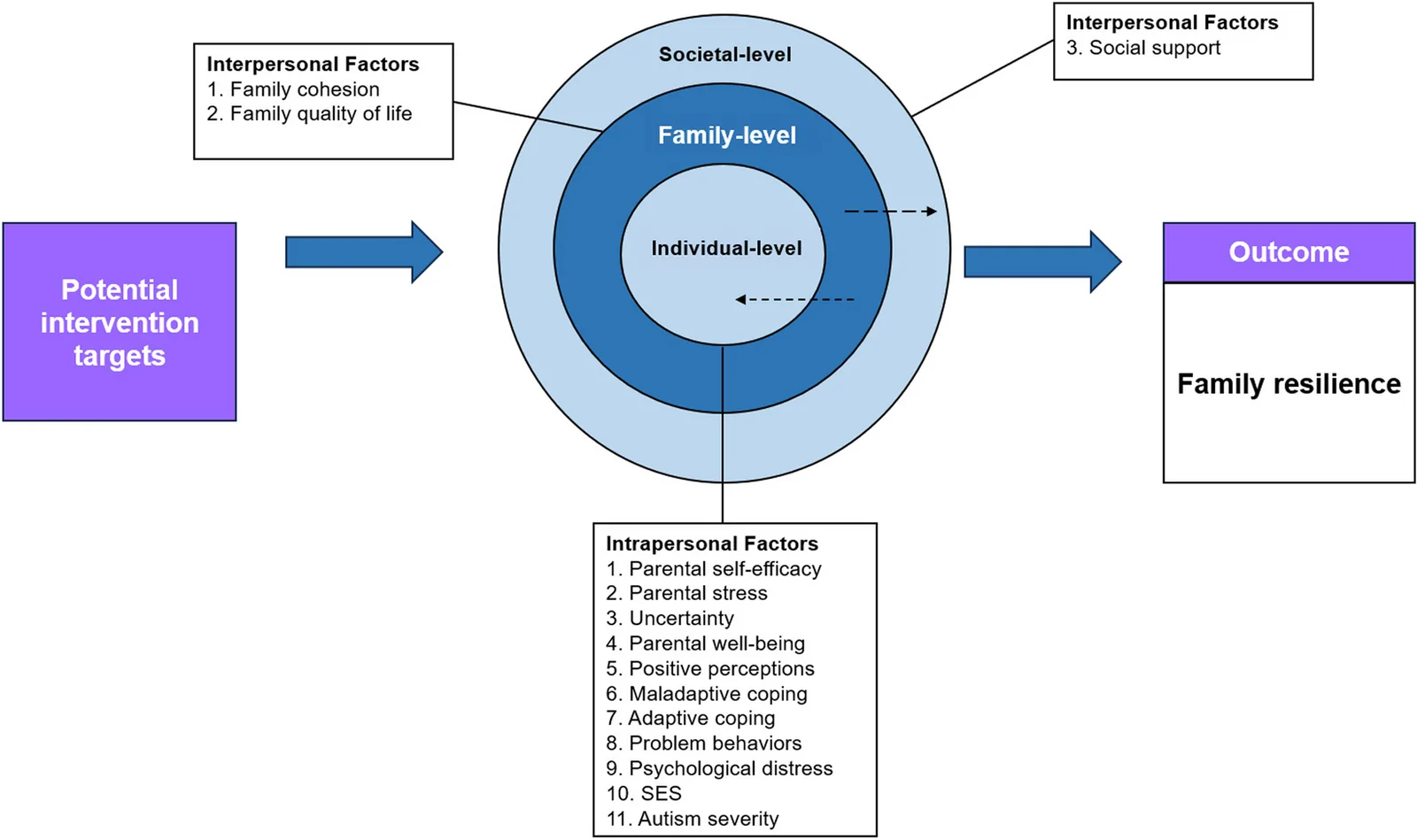
Integrated, multi-layered conceptual framework

Grounded in the findings of this meta-analysis, we propose an integrated, multi-layered conceptual framework to illuminate the relationships and mechanisms underlying resilience in families with autistic children (see Fig. 3). Consistent with Bronfenbrenner’s ecological perspective, the model treats the family as a complex network shaped by internal interactions and external influences, thereby capturing the systematic nature of family resilience [63].

This framework positions interpersonal/family‑level processes as the central mechanisms of family resilience, given that factors such as family cohesion and family quality of life demonstrated the strongest associations with family resilience. We propose that these core family interaction patterns function as the primary drivers within the family system. At the same time, the framework incorporates intrapersonal factors (e.g., parental self‑efficacy, reduced parental stress) and societal‑level factors (e.g., social support) as synergistic contributing factors. Furthermore, we theorize a “ripple‑effect” mechanism: optimized family processes may cascade downward to bolster individual members’ psychological resources (e.g., self‑efficacy) and outward to motivate more active seeking and utilization of social resources. In turn, these individual and societal gains may further consolidate positive family dynamics, forming a virtuous cycle. Together, these propositions indicate that family resilience is sustained through a systematic and dynamic interplay across multiple ecological levels.

### Practical implications

The proposed framework also provides valuable directions and intervention targets for enhancing family resilience within this population. First, given the primacy of interpersonal/family‑level processes, interventions should prioritize strengthening family cohesion, communication, and interaction. Specifically, practitioners could build family bonds and improve overall family quality of life by promoting regular family rituals (e.g., shared meals, bedtime games, family meetings, daily walks) and teaching effective communication. Second, these efforts should be deliberately combined with empowering individual caregivers (e.g., sharing effective parenting strategies to enhance parenting self‑efficacy and teaching stress management techniques to reduce parenting stress). Concurrently, practitioners should help parents mobilize both informal and formal social support, for instance, by facilitating peer support networks, connecting families with community resources, or providing family support navigators who assist in service coordination. Furthermore, considering the multi‑level nature of the framework, practitioners can use a brief, framework‑based assessment tool to systematically identify protective and risk factors at intrapersonal and interpersonal levels according to each family’s unique circumstances. This approach enables practitioners to pinpoint the areas most in need of improvement and to tailor interventions to each family’s specific needs. By taking a holistic approach, practitioners can enhance supportive family interactions and reinforce parents’ psychological resources, which can, in turn, promote their autistic children’s development. Given that the current evidence base is predominantly cross‑sectional, these practical implications should be considered preliminary and hypothesis-generating and interpreted with caution, and future research should employ longitudinal and experimental designs to test the proposed framework and its bidirectional pathways.

### Limitations

It is important to acknowledge that this study has several limitations. First, the included studies exhibited high heterogeneity, likely driven by variations in sample size (ranging from 34 to 3,247), study design, and measurement approaches. Although we used a random-effects model to account for between-study variability, this heterogeneity may still compromise the robustness and generalizability of our conclusions. To further explore the sources of this heterogeneity, we conducted moderator analyses (e.g., mean age, publication year, study region, and study quality), yet most failed to account for the substantial between-study variation. Only the sample size reached statistical significance, but the coefficient was small, and its substantive impact was limited, indicating minimal explanatory power. This largely unexplained heterogeneity likely reflects unmeasured study or sample characteristics—such as measurement instruments or cultural contexts—that were not captured by our moderator analysis. Accordingly, future studies should identify and incorporate additional moderators to yield more robust evidence. Overall, both the pooled estimates and the moderator findings should be interpreted with caution.

Second, the effect sizes presented in this study are obtained from the correlation coefficients of cross-sectional studies, which limits the ability to draw causal conclusions regarding the relationships between risk/protective factors and family resilience. Accordingly, the implications of these findings should be interpreted with caution. Future longitudinal research is needed to examine the proposed framework and potential bidirectionality of these relationships.

Third, to enable a unified synthesis of effect sizes, we converted ORs from two studies and *β* coefficients from five studies into Pearson’s *r* using established methods. This approach places different statistical indicators on a common metric, facilitating quantitative integration. However, such conversions rely on several statistical assumptions and yield approximations rather than exact equivalences [43]. Given heterogeneity across primary studies in analytic models, measurement instruments, and variable dichotomization, the converted *r* values may introduce methodological bias and potentially inflate heterogeneity. Therefore, these results should be interpreted with caution.

Fourth, some pooled estimates are based on a relatively small number of primary studies (e.g., parental self-efficacy and uncertainty). Therefore, these effect size estimates should be interpreted cautiously due to the limited evidence base.

Finally, it is essential to acknowledge that most of the study participants are female, which limits the generalizability of the findings to male caregivers. These limitations underscore the need for further studies to explore the issues and broaden the current knowledge of family resilience across a variety of different contexts.

## Conclusion

This study is the first systematic review and meta-analysis on the risk and protective factors for family resilience among families who have autistic children. Our results show that there were two categories of risk and protective factors, including interpersonal factors (e.g., family cohesion, social support) and intrapersonal factors (e.g., parental self-efficacy, uncertainty). Our findings revealed that interpersonal factors exhibited the strongest individual associations with family resilience compared to intrapersonal factors. In addition, we identified some salient (e.g., family cohesion) and contextually unique (e.g., uncertainty) risk and protective factors for family resilience among families with autistic children. These findings underscore the need for a systemic, multilevel framework to understand and support family resilience in this population. Building on this, we propose a multi-level family resilience conceptual framework that can inform targeted interventions for these families.

## Supplementary Information

Supplementary Material 1.

## Data Availability

All data generated or analyzed during this study are included in this published article.

## Abbreviations

ASD: Autism spectrum disorder
ADHD: Attention-deficit/hyperactivity disorder
PRISMA: Preferred reporting items for systematic reviews and meta-analyses
