# Supplementary material for: Towards a multi-level family resilience promotion framework: a meta-analysis of factors associated with family resilience in families of autistic children
Source: BMC Psychol. 2026 Jul 16;14:1465. doi: 10.1186/s40359-026-05162-5 (PMC13656514; doi:10.1186/s40359-026-05162-5)
Supplement: Supplementary file 1 [file 40359_2026_5162_MOESM1_ESM.docx]

**Supplementary Content**

**Supplementary Table 1:** Preferred Reporting Items for Systematic Reviews and Meta-Analyses (PRISMA)

**Supplementary Table 2:** Search terms

**Supplementary Table 3:** Characteristics of the included studies

**Supplementary Table 4:** Categorical moderators for certain risk/protective factors

**Supplementary Table 5:** Meta-regression results for continuous study characteristic and demographic moderators

# Supplementary Table 1: Preferred Reporting Items for Systematic Reviews and Meta-Analyses (PRISMA)

| **Section/topic** | **#** | **Checklist item** | **Reported on page #** |
| --- | --- | --- | --- |
| **TITLE** | | |  |
| Title | 1 | Identify the report as a systematic review, meta-analysis, or both. | Title page |
| **ABSTRACT** | | |  |
| Structured summary | 2 | Provide a structured summary including, as applicable: background; objectives; data sources; study eligibility criteria, participants, and interventions; study appraisal and synthesis methods; results; limitations; conclusions and implications of key findings; systematic review registration number. | 1-2 |
| **INTRODUCTION** | | |  |
| Rationale | 3 | Describe the rationale for the review in the context of what is already known. | 3-8 |
| Objectives | 4 | Provide an explicit statement of questions being addressed with reference to participants, interventions, comparisons, outcomes, and study design (PICOS). | 7-8 |
| **METHODS** | | |  |
| Protocol and registration | 5 | Indicate if a review protocol exists, if and where it can be accessed (e.g., Web address), and, if available, provide registration information including registration number. | 8 |
| Eligibility criteria | 6 | Specify study characteristics (e.g., PICOS, length of follow-up) and report characteristics (e.g., years considered, language, publication status) used as criteria for eligibility, giving rationale. | 8-9 |
| Information sources | 7 | Describe all information sources (e.g., databases with dates of coverage, contact with study authors to identify additional studies) in the search and date last searched. | 8 |

# Supplementary Table 1: (continued)

| **Section/topic** | **#** | **Checklist item** | **Reported on page #** |
| --- | --- | --- | --- |
| **METHODS** | | |  |
| Search | 8 | Present full electronic search strategy for at least one database, including any limits used, such that it could be repeated. | 8, Supplementary Table 2 |
| Study selection | 9 | State the process for selecting studies (i.e., screening, eligibility, included in systematic review, and, if applicable, included in the meta-analysis). | 8-9, Figure 1 |
| Data collection process | 10 | Describe method of data extraction from reports (e.g., piloted forms, independently, in duplicate) and any processes for obtaining and confirming data from investigators. | 9-10 |
| Data items | 11 | List and define all variables for which data were sought (e.g., PICOS, funding sources) and any assumptions and simplifications made. | 10 |
| Risk of bias in individual studies | 12 | Describe methods used for assessing risk of bias of individual studies (including specification of whether this was done at the study or outcome level), and how this information is to be used in any data synthesis. | 9 |
| Summary measures | 13 | State the principal summary measures (e.g., risk ratio, difference in means). | 11 |
| Synthesis of results | 14 | Describe the methods of handling data and combining results of studies, if done, including measures of consistency (e.g., I^2^) for each meta-analysis. | 11 |
| Risk of bias across studies | 15 | Specify any assessment of risk of bias that may affect the cumulative evidence (e.g., publication bias, selective reporting within studies). | 11 |

# Supplementary Table 1: (continued)

| **Section/topic** | **#** | **Checklist item** | **Reported on page #** |
| --- | --- | --- | --- |
| **METHODS** | | |  |
| Additional analyses | 16 | Describe methods of additional analyses (e.g., sensitivity or subgroup analyses, meta-regression), if done, indicating  which were pre-specified. | 12 |
| **RESULTS** | | |  |
| Study selection | 17 | Give numbers of studies screened, assessed for eligibility, and included in the review, with reasons for exclusions at each stage, ideally with a flow diagram. | 12 |
| Study characteristics | 18 | For each study, present characteristics for which data were extracted (e.g., study size, PICOS, follow-up period) and  provide the citations. | 12-13,  Supplementary Table 3 |
| Risk of bias within studies | 19 | Present data on risk of bias of each study and, if available, any outcome level assessment (see item 12). | Supplementary Table 3 |
| Results of individual studies | 20 | For all outcomes considered (benefits or harms), present, for each study: (a) simple summary data for each  intervention group (b) effect estimates and confidence intervals, ideally with a forest plot. | 13-14,  Table 1 |
| Synthesis of results | 21 | Present results of each meta-analysis done, including confidence intervals and measures of consistency. | Table 1, Figure 2 |
| Risk of bias across studies | 22 | Present results of any assessment of risk of bias across studies (see Item 15). | 15 |

# Supplementary Table 1: (continued)

| **Section/topic** | **#** | **Checklist item** | **Reported on page #** |
| --- | --- | --- | --- |
| **RESULTS** | | |  |
| Additional analysis | 23 | Give results of additional analyses, if done (e.g., sensitivity or subgroup analyses, meta-regression [see Item 16]). | Supplementary Table 4,5 |
| **DISCUSSION** | | |  |
| Summary of evidence | 24 | Summarize the main findings including the strength of evidence for each main outcome; consider their relevance to  key groups (e.g., healthcare providers, users, and policy makers). | 15-16 |
| Limitations | 25 | Discuss limitations at study and outcome level (e.g., risk of bias), and at review-level (e.g., incomplete retrieval of  identified research, reporting bias). | 23-24 |
| Conclusions | 26 | Provide a general interpretation of the results in the context of other evidence, and implications for future research. | 25 |
| Funding | 27 | Describe sources of funding for the systematic review and other support (e.g., supply of data); role of funders for the  systematic review. | Title page |

*From:* Moher D, Liberati A, Tetzlaff J, Altman DG, The PRISMA Group (2009). Preferred Reporting Items for Systematic Reviews and Meta-Analyses: The PRISMA Statement. PLoS Med 6(7): e1000097. doi:10.1371/journal.pmed1000097

For more information, visit: **www.prisma-statement.org**.

# Supplementary Table 2: Search terms

We applied broad search terms, with an example in Web of Science as follows:

| #ID | Query |
| --- | --- |
| #1 | child* (Topic) |
| #2 | autis* or autistic disorder or autism spectrum disorder or pervasive developmental disorder or Asperger* (Topic) |
| #3 | family resilienc* OR family hardiness OR family adaptation OR family adjustment (Topic) |

Example of Chinese search terms in China National Knowledge Infrastructure Database (CNKI) were as follows:

| #ID | Query |
| --- | --- |
| #1 | 自闭症+孤独症+儿童孤独症+幼儿自闭症+小儿自闭症 |
| #2 | 家庭抗逆力+家庭复原力+家庭韧性+家庭适应+家庭调整+家庭坚韧性 |

# Supplementary Table 3: Characteristics of the included studies

| **Study ID** | **Country** | **Age Mean (Range)** | **Female ratio** | **Autism status** | **Sample size** | **Measurement of the Family Resilience** | **Risk/protective factors** | **Category** | **Quality score** |
| --- | --- | --- | --- | --- | --- | --- | --- | --- | --- |
| Al-Jadiri et al. (2020) | USA | C:0-17 | C: L: 23.9; H:76.1 | Autism only | 1151 families | Indicator of Family Resilience in National Survey of Children’s Health (NSCH) | Parental factors, health care factors, child autism-related factors | Intrapersonal factor, interpersonal factor | 7 |
| Bristol (1987) | USA | C:5.3  (2.3-9.7) P:31 (19-47) | C:24.4 P:100 | Autism only | 45 mother and their autistic children | Home Quality Rating Scale (HQRS) | Severity of handicap, pile-up of stresses,  family cohesion, social support, self-blame, coping | Intrapersonal factor, interpersonal factor | 6 |
| Call (2021) | USA | C:12.23  (6-17)  P: 44.3 | P:72.5 | Autism only | 2091 parents | Indicator of Family Resilience in National Survey of Children’s Health (NSCH) | Problem behaviors, parental stress, parental mental health, | Intrapersonal factor | 7 |
| Cheatham and Fernando (2022) | USA | C:7.2  (2-12) P:37.3 | C:22.97 P:69 | Autism only | 71 parents | Family Resiliency Assessment Scale (FRAS) | Parental stress | Intrapersonal factor | 6 |

# Supplementary Table 3: (continued)

| **Study ID** | **Country** | **Age Mean (Range)** | **Female ratio** | **Autism status** | **Sample size** | **Measurement of the Family Resilience** | **Risk/protective factors** | **Category** | **Quality score** |
| --- | --- | --- | --- | --- | --- | --- | --- | --- | --- |
| Cripe (2013) | USA | C:0-17 | P: 50 | Autism only | 206 parents | Family Resiliency Assessment Scale (FRAS) | Father parental resilience, mother parental resilience, parent stress | Intrapersonal factor | 7 |
| Duca (2015) | Romania | C: 9.6  (4-17)  P:42.8  (27-53) | P:50 | Autism only | 100 parents | Family Resiliency Assessment Scale (FRAS) | Parenting stress, dyadic coping | Intrapersonal factor | 6 |
| Fan (2020) | China | C:68.41 (20-143) m  P:33.12 (23-61) | C:35  P:70 | Autism only | 80  caregivers | Family Hardiness Index (FHI) | Autism severity | Intrapersonal factor | 7 |
| Fong et al. (2021) | Canada | C:10.45  (2-18)  P:43.85  (27-64) | C:10.5  P: 80 | Autism co-occurring condition | 153 caregivers | Family Resilience Assessment Scale-ASD (FRAS-ASD) | Behavior problems,  satisfaction with informal supports | Intrapersonal factor, interpersonal factor | 8 |
| Gardiner (2014) | Canada | C:11.5  (6-18)  P:44.5  (30-65) | C:14.3  P: 90.5 | Autism co-occurring condition | 84 caregivers | Family Resilience Assessment Scale (FRAS) | FQOL, child age, family income, disability severity | Intrapersonal factor, interpersonal factor | 7 |

# Supplementary Table 3: (continued)

| **Study ID** | **Country** | **Age Mean (Range)** | **Female ratio** | **Autism status** | **Sample size** | **Measurement of the Family Resilience** | **Risk/protective factors** | **Category** | **Quality score** |
| --- | --- | --- | --- | --- | --- | --- | --- | --- | --- |
| Gardiner et al. (2019) | Canada | C: 11.93  P:45.32  (20-69) | C:11.5  P:83.9 | Autism only | 174 caregivers | Family Resiliency Assessment Scale (FRAS) | FQOL | Interpersonal factor | 6 |
| Gill and Harris (1991) | USA | C:9.9 (2-18)  P:38.8 (24-57) | C:21.7  P:100 | Autism only | 60 mothers | Family Hardiness Index (FHI) | Perceived social support | Interpersonal factor | 8 |
| Greeff and van der Walt (2010) | South Africa | C:6.48  P:36.21 (34-43) | C:8.8  P:70.6 | Autism only | 34 parents | Family Attachment and Changeability Index (FACI8) | Child age, SES, social support, passive appraisal | Intrapersonal factor, interpersonal factor | 6 |
| Hayes et al. (2022) | USA | C:10.05  (0-17)  P:43.11 | C:20.2  P:67.4 | Autism only | 654 caregivers | Indicator of Family Resilience in National Survey of Children’s Health (NSCH) | Community support, caregiver mental health | Intrapersonal factor, interpersonal factor | 7 |

# Supplementary Table 3: (continued)

| **Study ID** | **Country** | **Age Mean (Range)** | **Female ratio** | **Autism status** | **Sample size** | **Measurement of the Family Resilience** | **Risk/protective factors** | **Category** | **Quality score** |
| --- | --- | --- | --- | --- | --- | --- | --- | --- | --- |
| Higgins et al. (2022) | Ireland | C:10.5  (4-18)  P:42.58  (21-68) | C: 27.1  P: 85.3 | Autism co-occurring condition | 136 parents | Family Quality of Life Scale (FQOLS) | Self-injurious behavior frequency and severity, aggressive/destructive behavior frequency and severity, stereotyped behavior frequency, positive perceptions, self-efficacy, adaptive coping, social support, parenting stress, parental distress, psychological wellbeing | Intrapersonal factor, interpersonal factor | 8 |
| Hsiao (2024) | USA | C:3-17  P: NR | C: 19.84  P: NR | Autism only | 1003 parents | Indicator of Family Resilience in National Survey of Children’s Health (NSCH) | Parental stress | Intrapersonal factor | 5 |
| Hu et al. (2023) | China | C:0-14  P:≥ 18 | C:26.3  P: 81.4 | Autism only | 118 parents | Family Resilience Assessment Scale-Chinese version (FRAS-C) | Positive coping, negative coping | Intrapersonal factor | 8 |

# Supplementary Table 3: (continued)

| **Study ID** | **Country** | **Age Mean (Range)** | **Female ratio** | **Autism status** | **Sample size** | **Measurement of the Family Resilience** | **Risk/protective factors** | **Category** | **Quality score** |
| --- | --- | --- | --- | --- | --- | --- | --- | --- | --- |
| Jiang et al. (2024) | China | C:5.52  (3-14)  P:35.59  (19-53) | C: 19.6  P: 76.5 | Autism only | 260 parents | Family Resilience Assessment Scale-Chinese version (FRAS-C) | Caregiver burden | Intrapersonal factor | 8 |
| Kavaliotis (2017) | Greece | C:12.45  P: NR | C:18.9  P: 50 | Autism only | 624 parents | Family Resilience Assessment (FRAS) | Parental stress, social support | Intrapersonal factor, interpersonal factor | 6 |
| Kim et al. (2019) | USA | C:3-17  P:22-75 | C:18.9  P: 72.5 | Autism only | 1131 parents | Indicator of Family Resilience in National Survey of Children’s Health (NSCH) | Parenting stress | Intrapersonal factor | 7 |
| Lei and Kantor (2020) | China | C:9.73  (<18)  P: NR | C:32.90  P: NR | Autism  only | 168 parents | Chinese version of Family Adaptability and Cohesion Evaluation Scales (FACES II-CV). | The child’s functional level | Intrapersonal factor | 5 |
| Lei and Kantor (2022) | China | C:9.77  (2-17)  P: NR | C:33.30  P:56.40 | Autism only | 163 parents | Chinese version of Family Adaptability and Cohesion Evaluation Scales (FACES II-CV). | Social support, FQOL, family cohesion | Intrapersonal factor, interpersonal factor | 6 |

**Supplementary Table 3:** (continued)

| **Study ID** | **Country** | **Age Mean (Range)** | **Female ratio** | **Autism status** | **Sample size** | **Measurement of the Family Resilience** | **Risk/protective factors** | **Category** | **Quality score** |
| --- | --- | --- | --- | --- | --- | --- | --- | --- | --- |
| Li (2021) | China | C: 0-6  P: ≥18 | P:75 | Autism only | 168 parents | Family Hardiness Index (FHI) | Family management, family functioning |  | 6 |
| Lin et al. (2011) 1 | China (Taiwan） | C:17.17  P:46.58 (35-63） | C:15.8  P:100 | Autism only | 76 mother | The family adaptability and cohesion evaluation scales (FACES II) | Family cohesion, maternal depression, maternal anxiety, social support,  problem-focused coping, emotion-focused coping | Intrapersonal factor, interpersonal factor | 6 |
| Lin et al. (2011) 2 | USA | C:18  P:47.49 (32-73) | C:26.5  P:100 | Autism only | 325 mother | The family adaptability and cohesion evaluation scales (FACES II) | Family cohesion, maternal depression, maternal anxiety, social support,  problem-focused coping, emotion-focused coping | Intrapersonal factor, interpersonal factor | 7 |
| Manning et al. (2011) | USA | C:8.8(6-12）  P:40.9(24-54） | C:16.9  P:95.9 | Autism only | 195 caregivers | Family Environment Scale (FES) | Autism severity, problem behavior severity, stress, informal support, coping by relying on family and friends, coping by seeking spiritual support, reframing, subjective SES | Intrapersonal factor, interpersonal factor | 7 |

**Supplementary Table 3:** (continued)

| **Study ID** | **Country** | **Age Mean (Range)** | **Female ratio** | **Autism status** | **Sample size** | **Measurement of the Family Resilience** | **Risk/protective factors** | **Category** | **Quality score** |
| --- | --- | --- | --- | --- | --- | --- | --- | --- | --- |
| Meleady et al. (2020) | Ireland | C:9.1(4-17)  P:41.2(27-55) | C:26  P:94.2 | Autism only | 104 parents | Family Quality of Life Scale (FQOLS) | Autism symptom severity, problem behaviors, pile-up life demands, social support, positive contributions, problem-focused coping, emotional-approach coping, passive-avoidance coping, satisfaction with life | Intrapersonal factor, interpersonal factor | 8 |
| Menezes et al. (2024) | USA | C:11.32  (<18)  P: NR | C:19.86  P:72.62 | Autism only | 3247 parents | Indicator of Family Resilience in National Survey of Children’s Health (NSCH) | Neighborhood support | Interpersonal factor | 5 |
| O'Brien (2014) | USA | C:16.2  P:49.3(39-67) | C:15.5  P:91.3 | Autism only | 103 caregivers | The General Functioning subscale (GF), derived from the McMaster Family Assessment Device (FAD), | Behavior problems, daily stressors, uncertainty, coping strategies | Intrapersonal factor | 8 |

**Supplementary Table 3:** (continued)

| **Study ID** | **Country** | **Age Mean (Range)** | **Female ratio** | **Autism status** | **Sample size** | **Measurement of the Family Resilience** | **Risk/protective factors** | **Category** | **Quality score** |
| --- | --- | --- | --- | --- | --- | --- | --- | --- | --- |
| O'Brien (2016) | USA | C:16.2  P:49.3(39-67) | C:15.5  P: NR | Autism only | 103 caregivers | The General Functioning subscale (GF), derived from the McMaster Family Assessment Device (FAD), | Coping strategies | Intrapersonal factor | 8 |
| Paynter et al. (2013) | Australian | C:2.5-6  P:37.4 | C:18.6  P:58.1 | Autism only | 43 parents | The Impact on Family Scale (IFS) | Symptom severity, pile-up demands, internal resources, external resources, coping strategies, appraisal of stressor | Intrapersonal factor, interpersonal factor | 5 |
| Plumb (2011) | USA | C:6-12  P: NR | C:16.0  P:94.0 | Autism co-occurring condition | 50 parents | Family Resilience Assessment Scale (FRAS) | Parental stress | Intrapersonal factor | 5 |
| Simelane (2015) | South Africa | C:7.68(1-18)  P:39.01(22-64) | C: NR  P:82.4 | Autism only | 102 parents | Family Resilience Assessment Scale (FRAS) | Parental stress | Intrapersonal factor | 6 |

**Supplementary Table 3:** (continued)

| **Study ID** | **Country** | **Age Mean (Range)** | **Female ratio** | **Autism status** | **Sample size** | **Measurement of the Family Resilience** | **Risk/protective factors** | **Category** | **Quality score** |
| --- | --- | --- | --- | --- | --- | --- | --- | --- | --- |
| Spielfogel (2023) | USA | C:NR  P:47.26(28-71) | C:NR  P:86.7 | Autism only | 105 parents | Family Resilience Assessment Scale (FRAS) | FQOL, social resources, meaning-making | Intrapersonal factor, interpersonal factor | 6 |
| Van Ormer  (2020) | USA | C:12.10(6-17)  P:44.96 | C:19.1  P:72.3 | Autism only | 1091 parents | Indicator of Family Resilience in National Survey of Children’s Health (NSCH) | Parental stress,  parental mental health, formal support, peer support | Intrapersonal factor, interpersonal factor | 5 |
| Warter (2009) | USA | C:10.4  P:42.6(25-69) | C:18.8  P:90.8 | Autism co-occurring condition | 207 parents | Family Quality of Life Survey (FQOL) | Optimism, autism severity,  uncertainty | Intrapersonal factor | 6 |
| Weiss et al. (2013) | Canada | C:13.13  P:44.28(31-71) | C:15.9  P:100 | Autism co-occurring condition | 138 mothers | Family hardiness (IFPF) | Stressors, social support, self-efficacy, child age | Intrapersonal factor, interpersonal factor | 8 |
| Wong et al. (2020) | USA | NR | C:NR  P:68.2 | Autism only | 226 caregivers | The Beach Center Family Quality of Life Scale  (FQOL) | Emotion-focused coping, problem-focused coping | Intrapersonal factor | 6 |

**Supplementary Table 3:** (continued)

| **Study ID** | **Country** | **Age Mean (Range)** | **Female ratio** | **Autism status** | **Sample size** | **Measurement of the Family Resilience** | **Risk/protective factors** | **Category** | **Quality score** |
| --- | --- | --- | --- | --- | --- | --- | --- | --- | --- |
| Wu et al. (2022) | China | C:5.59 (3-14)  P:35.87 (19-64) | C:19.6  P:76.5 | Autism only | 260 parents | The Family Resilience Assessment Scale (FRAS) | Caregiver burden, social support | Intrapersonal factor, interpersonal factor | 8 |
| Wu et al. (2024) | China | C:5.23 (2-7)  P:35.28 (23-48) | C: 27.3  P: 100 | Autism only | 205 mothers | Family Hardiness Index (FHI) | Psychological resilience | Intrapersonal factor | 6 |
| Xu et al. (2017) | China | C:37.78 (28-60m)  P:32.85 (24-60) | C:20.11  P:75.13 | Autism only | 189 caregivers | Family Hardiness Index (FHI) | Parental stress | Intrapersonal factor | 6 |
| Xue et al. (2014) | Singapore | C:6.9 (3.2–11.8)  M:37.9,  F: 42.2, (31-50) | C:7.7  P:70.8 | Autism co-occurring condition | 65 parents | The Family Adaptability and Cohesion Evaluation Scales IV (FACES IV) | Age of child, perceived stress, positive meanings | Intrapersonal factor, interpersonal factor | 5 |
| Zeng et al. (2019) | China | C:6.73(3-16)  P: NR | C:15.5  P: NR | Autism only | 303 parents | Family Resilience Assessment Scale (FRAS) | Social support, coping | Intrapersonal factor, interpersonal factor | 8 |

# Note: *C*: children; *F*: fathers; *M*: mothers; *NR:* not reported; *P*: parents

# Supplementary Table 4: Categorical moderators for certain risk/protective factors

| **Risk/protective factors** | **Categorical moderators** | | **N** | **Effect size r [95% CI]** | **Q** | **P-value** |
| --- | --- | --- | --- | --- | --- | --- |
| Parental stress | Study quality |  |  |  | 0.78 | 0.38 |
|  |  | Low | 3 | -0.40 [-0.52, -0.25] |  |  |
|  |  | Moderate & High | 8 | -0.49 [-0.62, -0.32] |  |  |
|  |  |  |  |  |  |  |
| Social support | Study region |  |  |  | 0.39 | 0.53 |
|  |  | Asian | 4 | 0.36 [0.27, 0.45] |  |  |
|  |  | Non-Asian | 16 | 0.40 [0.32, 0.48] |  |  |
|  | Study quality |  |  |  | 0.45 | 0.50 |
|  |  | Low | 3 | 0.32 [0.08, 0.53] |  |  |
|  |  | Moderate & High | 17 | 0.41 [0.32, 0.48] |  |  |
| Adaptive coping | Study region |  |  |  | 0.36 | 0.58 |
|  |  | Asian | 3 | 0.27 [0.16, 0.37] |  |  |
|  |  | Non-Asian | 10 | 0.31 [0.21, 0.41] |  |  |

# Note: In the factors of parental stress and adaptive coping, the number of studies in the study region and study quality, respectively, is less than three, making it impossible to include them in the moderator analysis.

# Supplementary Table 5: Meta-regression results for continuous study characteristic and demographic moderators

| **Risk/protective factors** | **Continuous moderator** | **β** | **95% CI** | **P-value** |
| --- | --- | --- | --- | --- |
| Parental stress |  |  |  |  |
|  | Sample size | 0.0002 | [0.00, 0.00] | 0.03 |
|  | Publication year | -0.01 | [-0.05, 0.03] | 0.49 |
|  | Mean age | 0.03 | [-0.07, 0.13] | 0.58 |
|  |  |  |  |  |
| Social support |  |  |  |  |
|  | Sample size | -0.00 | [-0.00, 0.00] | 0.30 |
|  | Publication year | -0.00 | [-0.01, 0.01] | 0.51 |
|  | Mean age | -0.02 | [-0.05, 0.00] | 0.05 |
| Adaptive coping |  |  |  |  |
|  | Sample size | -0.00 | [-0.00, 0.00] | 0.14 |
|  | Publication year | 0.02 | [-0.01, 0.04] | 0.21 |
|  | Mean age | -0.01 | [-0.03, 0.02] | 0.64 |

**Reference List:**

Al-Jadiri, A., Tybor, D. J., Mulé, C., & Sakai, C. (2020). Factors Associated with Resilience in Families of Children with Autism Spectrum Disorder. *Journal of Developmental & Behavioral Pediatrics*, 42(1), 16–22. <https://doi.org/10.1097/dbp.0000000000000867>

Bristol, M. M. (1987). Mothers of children with autism or communication disorders: Successful adaptation and the double ABCX model. *Journal of Autism and Developmental Disorders*, 17(4), 469–486. <https://doi.org/10.1007/bf01486964>

Call, T. A. (2021). *Parent Health Outcomes and the Role of Parent Stress and Family Resilience in Families of Children with Autism Spectrum Disorder.* The Florida State University.

Cheatham, K. L., & Fernando, D. M. (2021). Family Resilience and Parental Stress in Families of Children with Autism. *The Family Journal*, 30(3), 419–426. <https://doi.org/10.1177/10664807211052494>

Cripe, C. T. (2013). *Family resilience, parental resilience and stress mediation in families with autistic children*. Northcentral University.

Duca, D. S. (2015). Family resilience and parental stress: the effects on marital relationship in the context of a child diagnosed with an autism spectrum disorder. *Analele Ştiinţifice ale Universităţii» Alexandru Ioan Cuza «din Iaşi. Psihologie,* (1), 71-90.

Fan, Q. (2020). Analysis of influencing factors and nursing interventions for family resilience in primary caregivers of children with autism spectrum disorder. *Nursing Practice and Research*, 21, 30–32.

Fong, V., Gardiner, E., & Iarocci, G. (2020). Satisfaction with informal supports predicts resilience in families of children with autism spectrum disorder. *Autism*, 25(2), 452–463. <https://doi.org/10.1177/1362361320962677>

Gardiner, E. C. (2014). *Quality of life in families of children with autism spectrum disorder: Considerations of risk and resilience*. University of Victoria.

Gardiner, E., Mâsse, L. C., & Iarocci, G. (2019). A psychometric study of the Family Resilience Assessment Scale among families of children with autism spectrum disorder. *Health and Quality of Life Outcomes*, 17(1). <https://doi.org/10.1186/s12955-019-1117-x>

Gill, M. J., & Harris, S. L. (1991). Hardiness and social support as predictors of psychological discomfort in mothers of children with autism. *Journal of Autism and Developmental Disorders*, 21(4), 407-416. <https://doi.org/10.1007/BF02206867>

Greeff, A. P., & Van der Walt, K. J. (2010). Resilience in families with an autistic child. *Education and Training in Autism and Developmental Disabilities, 45*(3), 347-355.

Hayes, K. N., Rossetti, K. G., & Zlomke, K. (2022). Community support, family resilience and mental health among caregivers of youth with autism spectrum disorder. *Child: Care, Health and Development*, 49(1), 130–136. <https://doi.org/10.1111/cch.13025>

Higgins, L., Mannion, A., Chen, J. L., & Leader, G. (2022). Adaptation of Parents Raising a Child with ASD: The Role of Positive Perceptions, Coping, Self-efficacy, and Social Support. *Journal of Autism and Developmental Disorders*, 53(3), 1224–1242. <https://doi.org/10.1007/s10803-022-05537-8>

Hsiao, Y.-J. (2024). Parental stress, family resilience, and health-related quality of life: parents of children with autism spectrum disorder. *Current Psychology*, 43(39), 31019–31026. <https://doi.org/10.1007/s12144-024-06687-x>

Hu, N., Zhao, S., Xu, N. A., Wu, Z. W., & Cheng, X. N. (2023). Fear of disease progression and its influencing factors in parents of children with autism: A cross-sectional study. *Modern Nurse*, 8, 118–121.

Jiang, S. S., Tao, J. R., Wu, Y. H., Cen, Y. B. N., Huang, L. Q., & Dong, C. Q. (2024). Latent classes and influencing factors of caregiving burden among parents of children with autism. *Journal of Wenzhou Medical University*, 6, 438–445.

Kavaliotis, P. (2017). Investigation of the Correlation of Family Resilience of Parents with a Child with Autism Spectrum Disorders, Parenting Stress and Social Support. *International Journal of Psychological Studies*, 9(2), 1. <https://doi.org/10.5539/ijps.v9n2p1>

Kim, I., Dababnah, S., & Lee, J. (2019). The Influence of Race and Ethnicity on the Relationship between Family Resilience and Parenting Stress in Caregivers of Children with Autism. *Journal of Autism and Developmental Disorders*, 50(2), 650–658. <https://doi.org/10.1007/s10803-019-04269-6>

Lei, X., & Kantor, J. (2020). Social support and family quality of life in Chinese families of children with autism spectrum disorder: the mediating role of family cohesion and adaptability. *International Journal of Developmental Disabilities*, 68(4), 454–461. <https://doi.org/10.1080/20473869.2020.1803706>

Lei, X., & Kantor, J. (2020). Study on Family Cohesion and Adaptability of Caregivers of Children with ASD and Its Influencing Factors. *Социальная Психология и Общество*, 11(3), 70–85. <https://doi.org/10.17759/sps.2020110305>

Li, L., Wang, F. J., Liu, C. L., Zou, W., & Yang, X. D. (2021). Current status and influencing factors of family management in families of children with autism spectrum disorder. *Journal of Clinical Nursing*, 6, 23–26.

Lin, L.-Y., Orsmond, G. I., Coster, W. J., & Cohn, E. S. (2011). Families of adolescents and adults with autism spectrum disorders in Taiwan: The role of social support and coping in family adaptation and maternal well-being. *Research in Autism Spectrum Disorders*, 5(1), 144–156. <https://doi.org/10.1016/j.rasd.2010.03.004>

Manning, M. M., Wainwright, L., & Bennett, J. (2010). The Double ABCX Model of Adaptation in Racially Diverse Families with a School-Age Child with Autism. *Journal of Autism and Developmental Disorders*, 41(3), 320–331. <https://doi.org/10.1007/s10803-010-1056-1>

Meleady, J., Nearchou, F., Bramham, J., & Carr, A. (2020). Family adaptation among parents of children on the autism spectrum without a comorbid intellectual disability: A test of the Double ABCX model. *Research in Autism Spectrum Disorders*, 78, 101637. <https://doi.org/10.1016/j.rasd.2020.101637>

Menezes, M., Soland, J., & Mazurek, M. O. (2023). Association Between Neighborhood Support and Family Resilience in Households with Autistic Children. *Journal of Autism and Developmental Disorders*, 54(5), 1765–1773. <https://doi.org/10.1007/s10803-023-05951-6>

O’Brien, S. (2016). Families of Adolescents with Autism: Facing the Future. *Journal of Pediatric Nursing*, 31(2), 204–213. <https://doi.org/10.1016/j.pedn.2015.10.019>

O’Brien, S. L. (2013). *The Influence of Daily Stressors, Severity of Behavior Problems, Uncertainty, and Coping Strategies on Family Adaptation in Families of Adolescents with Autism Spectrum Disorders*. The Catholic University of America.

Paynter, J., Riley, E., Beamish, W., Davies, M., & Milford, T. (2013). The double ABCX model of family adaptation in families of a child with an autism spectrum disorder attending an Australian early intervention service. *Research in Autism Spectrum Disorders*, 7(10), 1183–1195. <https://doi.org/10.1016/j.rasd.2013.07.006>

Plumb, J. C. (2011). The impact of social support and family resilience on parental stress in families with a child diagnosed with an autism spectrum disorder. *Social Work*, 34(6), 124-135.

Simelane, A. (2015). *The role of resilience and socio-economic status in the parenting of children with autism spectrum disorder in South Africa*. Wits University.

Spielfogel, S. A. (2023). *Resilience in Families with Children on the Autism Spectrum: Meaning-Making and Social Support.* Alliant International University.

VanOrmer, J. (2020). *Parental Mental Health in Children with Autism: Effects of Parental Support, Family Resilience, and Parental Stress*. University of South Alabama.

Warter, E. H. (2009). *Promoting resiliency in families of individuals diagnosed with an autism spectrum disorder: The relationship between parental beliefs and family adaptation*. Boston College.

Weiss, J. A., Robinson, S., Fung, S., Tint, A., Chalmers, P., & Lunsky, Y. (2013). Family hardiness, social support, and self-efficacy in mothers of individuals with Autism Spectrum Disorders. *Research in Autism Spectrum Disorders*, 7(11), 1310–1317. <https://doi.org/10.1016/j.rasd.2013.07.016>

Wong, V., McGrew, J., & Ruble, L. (2020). Predicting the Outcomes of Parents of Transition-Age Youth or Young Adults with ASD. *Journal of Autism and Developmental Disorders*, 50(8), 2723–2739. <https://doi.org/10.1007/s10803-020-04362-1>

Wu, Y. H., Dong, C. Q., Cen, Y. B. N., Huang, L. Q., Chen, S. Y., & Yu, K. Y. (2022). Family resilience and its influencing factors in families of children with autism spectrum disorder. *Military Nursing*, 8, 34–37.

Wu, S. Q., Yang, N. B., Yuan, X. Q., Lin, R. F., Wu, S. H., Liu, L. Q., & Li, Z. Z. (2024). The mediating role of family resilience between psychological resilience and disease benefit perception in mothers of preschool children with autism. *Chinese Nursing Management*, 2, 315–320.

Xu, Y. Y., Liu, G. H., Li, G. K., Ou, P., Qian, C. F., Yang, S. W., & Qian, X. F. (2017). Family resilience and its influencing factors among caregivers of children with autism spectrum disorder. *Journal of Nursing Science*, 7, 65–68.

Xue, J., Ooh, J., & Magiati, I. (2013). Family functioning in Asian families raising children with autism spectrum disorders: the role of capabilities and positive meanings. *Journal of Intellectual Disability Research*, 58(5), 406–420. <https://doi.org/10.1111/jir.12034>

Zeng, Y., Ou, J. J., Yang, J. X., & Li, Y. M. (2019). Current status and influencing factors of family resilience in children with autism spectrum disorder. *Chinese Journal of Clinical Psychology*, 27(6), 1271–1274.
